# The association between dietary fat and mineral intake with semen parameters: A cross-sectional study in infertile men

**DOI:** 10.18502/ijrm.v20i5.11053

**Published:** 2022-06-08

**Authors:** Mehran Nouri, Sanaz Mehrabani, Hossein Firoozbakht, Elmira Vataniyan, Homayoun Abbasi, Mahsa Shirani

**Affiliations:** ^1^Students' Research Committee, School of Nutrition and Food Science, Shiraz University of Medical Sciences, Shiraz, Iran.; ^2^Department of Community Nutrition, School of Nutrition and Food Science, Shiraz University of Medical Sciences, Shiraz, Iran.; ^3^Department of Community Nutrition, School of Nutrition and Food Science, Food Security Research Center, Isfahan University of Medical Sciences, Isfahan, Iran.; ^4^Department of Nutrition, Science and Research Branch, Islamic Azad University, Tehran, Iran.; ^5^Department of Cellular and Molecular Nutrition, School of Nutritional Science and Dietetics, Tehran University of Medical Science, Tehran, Iran.; ^6^Isfahan Fertility and Infertility Center, Isfahan, Iran.; ^7^Students' Research Committee, Isfahan University of Medical Sciences, Isfahan, Iran.

**Keywords:** Infertility, Semen quality, Minerals, Fats, Cross-sectional study.

## Abstract

**Background:**

Infertility has been a significant problem for couples in recent decades, and the issue can often lie with the male rather than the female.

**Objective:**

This study aimed to investigate the effects of fats and minerals intake on semen parameters in infertile men.

**Materials and Methods:**

This cross-sectional study was performed on 260 infertile men referred to Isfahan Infertility Clinic, Isfahan, Iran in the summer of 2018. Semen parameters regarding sperm concentration, total motility, normal morphology, and sperm volume were considered. To assess dietary intakes, a validated food frequency questionnaire was used.

**Results:**

In the adjusted model, the prevalence of abnormal concentration was 5.23 times higher in the top quartile of calcium intake, compared with the bottom quartile (p = 0.03). Also, the prevalence of abnormal morphology was 68% lower in the third quartile of calcium intake (p = 0.03). Additionally, the prevalence of abnormal concentration was 84% lower in the top quartile of folate intake in comparison to the bottom quartile (p = 0.01) and the prevalence of abnormal morphology was 70% lower in the top quartile of folate intake (p = 0.03). Additionally, the prevalence of abnormal concentration was 72% lower in the top quartile of selenium intake in comparison to the bottom quartile (p = 0.04). Furthermore, in the crude model, the prevalence of abnormal volume was 64% lower in the second quartile of linoleic acid intake rather than the first quartile (p = 0.01).

**Conclusion:**

In conclusion, diets containing higher amounts of folate and selenium, and lower amounts of cholesterol, saturated fatty acid and calcium were associated with more favorable semen quality parameters.

## 1. Introduction

Over recent decades, infertility has been a significant issue in developing countries, affecting 15% of all couples falling within fertile age brackets (1). A meta-analysis estimated the infertility rate within the Iranian population to be about 10.9%. In approximately 40% of infertile couples, male infertility is a factor (2). Infertility is defined as the inability of a couple to conceive within 12 months or more of regular unprotected intercourse (3).

Infertility can be caused by various disorders including: anatomical or hormonal problems, genetic abnormalities, and infections. In addition, environmental factors such as air pollution, industrial chemicals, stress and depression, alcohol consumption, or smoking are all considered to be risk factors, which reduce sperm quality parameters in developed countries (4). Moreover, studies have shown a relationship between semen quality and lifestyle factors, including physical activity and diet (5).

Dietary fats are one of the dietary components that can contribute to infertility. Due to large amounts of unsaturated fatty acids in the membrane of sperm cells, the chance of lipid peroxidation in sperm cells and production of free radicals such as hydrogen peroxide, superoxide, and hydroxyl radicals is high (6, 7). Lipid peroxidation can drastically alter membrane integrity and permeability, alter cellular enzyme activation and cell apoptosis, and thus affect spermatozoa. As a result, sperm count and motility decrease and sperm shapes become abnormal (8).

Furthermore, over-consumption of saturated and trans-fatty acids can reduce sperm quality by increasing the rate of inflammation (9). Meanwhile, previous studies have shown that there is an important association between the intake of certain dietary minerals, zinc and iron, and semen quality (10-12). Although the association between dietary fat and the quality of sperm has been evaluated before, the results are controversial.

Therefore, this study aimed to evaluate the association between dietary fats and minerals with factors related to semen quantity and quality.

## 2. Materials and Methods

### Study design

This cross-sectional study was performed on 260 infertile men referred to Isfahan Infertility Clinic in Isfahan, Iran, in June-August 2018. Participants were aged between 18-55 yr with a history of primary or secondary infertility in the last 5 yr. All participants voluntarily entered the study. Exclusion criteria were: a history of urinary infection, use of supplements, androgens, anticoagulants, cytotoxic drugs or immunosuppressant, and metabolic diseases such as diabetes or renal disease (13). Additionally, participants with incomplete information or caloric intakes outside the range of 800-4200 kcal/day were excluded from the study. Finally, the data for 260 cases were used in the study.

### Sperm parameters

After 3 days of abstinence, semen samples were taken from participants. The samples were collected in sterile containers and placed at room temperature for 30 min to ensure liquidity. Analysis of the semen samples was done according to the 5 ${}^{\text{th}}$ edition of the World Health Organization laboratory manual for human semen (14). 4 dependent semen parameters were measured: sperm concentration (SC); total sperm movement (TSM); normal sperm morphology (NSM); and seminal volume (SV). Abnormal semen parameters were defined as oligospermia: SC < 20 M/ml, TSM < 60%, sperm volume < 3 ml and NSM < 65% (13). As we were not able to analyze data with NSM < 65% in our study, the World Health Organization cut point of NSM < 4% was considered for normal morphology (13).

### Assessment of dietary intakes

To assess dietary intakes of individuals, a validated detailed food frequency questionnaire was used in this study. It included 168 food items comprising the most common Iranian foods and has been previously shown to be valid and reproducible for usein Iranian adults (15).

For each food item, participants reported their average consumption during the last year in terms of frequency of use. This classification includes the following: ≥ 6 times a day, 3-5 times a day, 2-3 times a day, once a day, 5-6 times a week, 2-4 times a week, once a week, 1-3 times a month and < 1 a month. The selected group was then converted to the daily intake for each food item. Nutritionist IV for Windows software was used to determine micronutrient intakes for each participant.

### Assessment of other variables

Demographic data, medical history, and intake of supplements were collected via a structured questionnaire for all participants. In this study, weight and height were measured with high accuracy and then body mass index was calculated according to the formula (kg/m^2^).

### Ethical considerations

The study was ethically approved by the Ethical Committee of Isfahan University of Medical Sciences, Isfahan, Iran (Code: IR.MUI.RESEARCH.REC.1397.232). All of the participants completed the informed consent form.

### Statistical analysis

Statistical analyses were carried out using the Statistical Package for the Social Sciences (SPSS) for Windows software (version 20.0, SPSS Inc, Chicago, IL, USA). P-values < 0.05 were considered statistically significant. To describe quantitative variables, mean ± standard deviation (SD) and for the categorical outcome, frequency and percentage were used. Analysis of covariance (ANCOVA) was used to determine the association between semen parameters as dependent variables, and fats and minerals covariates as independent variables.

Multiple logistic regression (odds ratios [ORs] with 95% confidence intervals [CI]) was used to evaluate the relationship between dietary fat and mineral intakes with sperm quality parameters in the crude and adjusted models. In the adjusted model, potential confounding variables including age, BMI, education, total energy intake, alcohol, smoking, and vitamin-mineral use were justified by ANCOVA, and the first quartile was considered as the reference level.

## 3. Results

###  Baseline characteristics of participants

Finally, 260 participants aged between 18 and 55 yr were included in this study. 36% of the subjects were smokers, and 20% of participants used alcohol. Furthermore, 30% of participants used multivitamins. All of the participants had semen analyses performed and the semen analysis parameters included: the mean SC; SV; TSM; and NSM. Also, the dietary intake of participants is shown in table I.

### Correlation between sperm related parameters and dietary components

There was a significant association between dietary intakes of protein and sperm volume (p < 0.001), dietary intakes of carbohydrates and SC (p = 0.01), and also dietary intakes of carbohydrates and sperm motility (p = 0.03, Table II).

### Association between dietary mineral intakes and sperm parameters

In the adjusted model, the prevalence of abnormal SC was 5.23 times higher in the top quartile of calcium intake, compared with the bottom quartile (p = 0.03). Also, the prevalence of abnormal sperm morphology was 68% lower in the third quartile of calcium intake, compared with the first quartile (p = 0.03). Additionally, the prevalence of abnormal SC was 84% lower in the top quartile of folate intake in comparison to the bottom quartile (p = 0.007) and the prevalence of abnormal sperm morphology was 70% lower in the top quartile of folate intake, compared with the first quartile (p = 0.03). Also, the prevalence of abnormal SC was 72% lower in the top quartile of selenium intake in comparison to the bottom quartile (p = 0.04, Table III).

### Association between dietary fat intakes and sperm parameters

As is shown in the adjusted model, the prevalence of abnormal SC was 5.6 and 4.8 times higher in the third quartile of cholesterol and saturated fatty acid (SFA) intake, respectively, compared with the first quartile (p = 0.01). Additionally, the prevalence of abnormal sperm volume was 64% lower in the second quartile of linoleic acid intake than in the first quartile (p = 0.01) in the crude model; however, this was not significant in the adjusted model (Table IV).

**Table I:** Baseline characteristics of participants

**Characteristics**	**Mean ± SD (n = 260)**
**Age (yr)**	31.24 ± 4.23
**BMI (kg/m**2**)**	26.94 ± 4.09
**MET (MET-h/wk)**	29.27 ± 2.12
**WC (cm)**	94.51 ± 10.35
**Dietary intake**
	**Energy (kcal)**	2516.51 ± 686.94
	**Carbohydrate (gr/day)**	361.38 ± 104.45
	**Protein (gr/day)**	94.07 ± 27.04
	**Fat (gr/day)**	86.77 ± 36.48
	**Caffeine (mg/day)**	79.41 ± 61.05
**Semen parameters**
	**SV (mL)**	4.13 ± 2.06
	**SC (mill/mL)**	13.11 ± 6.01
	**TSM (%)**	29.76 ± 18.08
	**NSM (%)**	2.00 ± 1.68

Data presented as Mean + SD. BMI: Body mass index, MET: Metabolic equivalents, WC: Waist circumference, SV: Semen volume, SC: Sperm concentration, TSM: Total sperm motility, NSM: Normal sperm morphology

**Table II:** Correlation between sperm-related parameters and dietary components (n = 260)

**Mean range of value**	**Calorie intake (kcal)**	**Protein (gr/day)**	**Carbohydrate (gr/day)**	**Fat (gr/day)**	**Caffeine**
**SV (mL)**	0.10 (0.08)	0.18 (0.00)	0.08 (0.19)	0.10 (0.10)	0.06 (0.29)
**SC (10**6**/ml)**	0.07 (0.24)	0.02 (0.64)	0.14 (0.01)	-0.05 (0.37)	0.03 (0.56)
**TSM (%)**	0.05 (0.34)	-0.02 (0.72)	0.13 (0.03)	-0.05 (0.34)	0.05 (0.37)
**NSM (%)**	-0.03 (0.61)	-0.09 (0.12)	0.00 (0.98)	-0.05 (0.36)	0.05 (0.37)

Values are reported by correlation coefficient (p-value) as assessed by regression. SV: Semen volume, SC: Sperm concentration, TSM: Total sperm motility, NSM: Normal sperm morphology

**Table III:** Abnormal semen quality across quartiles of mineral intake (n = 260)

**Variables**	**SC ˂ 20 M/ml**	**P-value**	**TSM ˂ 60%**	**P-value**	**NSM ˂ 4%**	**P-value**	**SV ˂ 3 ml**	**P-value**
**Calcium**
	**Crude**
		**Q1**	Reference		Reference		Reference		Reference	
		**Q2**	1.00 (0.38-2.59)	1.00	0.65 (0.10-4.06)	0.65	0.66 (0.27-1.62)	0.36	1.07 (0.50-2.27)	0.84
		**Q3**	1.13 (0.42-2.99)	0.80	0.31 (0.06-1.60)	0.16	0.40 (0.17-0.96)	0.04	1.23 (0.59-2.60)	0.57
		**Q4**	2.18 (0.70-6.78)	0.17	0.48 (0.08-2.74)	0.41	1.13 (0.42-2.99)	0.80	0.66 (0.29-1.47)	0.31
	**Adjusted**
		**Q1**	Reference		Reference		Reference		Reference	
		**Q2**	1.41 (0.50-3.99)	0.51	1.17 (0.17-8.04)	0.87	0.58 (0.22-1.50)	0.26	1.19 (0.53-2.66)	0.66
		**Q3**	1.95 (0.61-6.27)	0.25	0.74 (0.11-4.76)	0.75	0.32 (0.11-0.89)	0.03	1.45 (0.60-3.49)	0.40
		**Q4**	5.23 (1.13-24.25)	0.03	2.18 (0.22-21.32)	0.50	0.83 (0.24-2.90)	0.78	0.84 (0.30-2.40)	0.75
**Zinc**
	**Crude**
		**Q1**	Reference		Reference		Reference		Reference	
		**Q2**	0.40 (0.14-1.14)	0.08	0.18 (0.02-1.65)	0.13	0.60 (0.26-1.36)	0.22	1.32 (0.63-2.77)	0.45
		**Q3**	0.63 (0.21-1.89)	0.41	0.18 (0.02-1.65)	0.13	1.00 (0.42-2.36)	1.00	0.92 (0.43-1.98)	0.84
		**Q4**	1.00 (0.30-3.28)	1.00	0.23 (0.02-2.19)	0.20	1.78 (0.68-4.64)	0.23	0.72 (0.33-1.59)	0.42
	**Adjusted**
		**Q1**	Reference		Reference		Reference		Reference	
		**Q2**	0.49 (0.16-1.49)	0.21	0.35 (0.03-3.34)	0.36	0.72 (0.30-1.73)	0.46	1.49 (0.67-3.32)	0.32
		**Q3**	0.88 (0.24-3.13)	0.84	0.55 (0.05-5.72)	0.61	1.35 (0.48-3.76)	0.56	1.12 (0.45-2.76)	0.79
		**Q4**	1.78 (0.34-9.23)	0.48	1.85 (0.10-33.09)	0.67	3.04 (0.78-11.87)	0.10	1.01 (0.33-3.04)	0.97
**Folate**
	**Crude**
		**Q1**	Reference		Reference		Reference		Reference	
		**Q2**	0.84 (0.26-2.65)	0.77	0.00 (0.00)	0.99	1.00 (0.40-2.50)	1.00	1.00 (0.47-2.13)	1.00
		**Q3**	1.55 (0.41-5.77)	0.51	0.00 (0.00)	0.99	0.81 (0.33-1.98)	0.65	1.00 (0.47-2.13)	1.00
		**Q4**	0.28 (0.10-0.78)	0.01	0.00 (0.00)	0.99	0.53 (0.22-1.23)	0.14	0.92 (0.43-1.98)	0.84
	**Adjusted**
		**Q1**	Reference		Reference		Reference		Reference	
		**Q2**	0.67 (0.20-2.24)	0.52	0.00 (0.00)	0.99	0.78 (0.30-2.06)	0.62	1.15 (0.52-2.56)	0.72
		**Q3**	1.06 (0.25-4.44)	0.93	0.00 (0.00)	0.99	0.54 (0.19-1.49)	0.23	1.31 (0.55-3.12)	0.53
		**Q4**	0.16 (0.04-0.60)	0.007	0.00 (0.00)	0.99	0.30 (0.09-0.91)	0.03	1.48 (0.55-3.12)	0.43
**Selenium**
	**Crude**
		**Q1**	Reference		Reference		Reference		Reference	
		**Q2**	2.70 (1.03-7.11)	0.04	1.00 (0.19-5.14)	1.00	0.90 (0.36-2.21)	0.81	0.85 (0.39-1.860	0.69
		**Q3**	2.32 (0.91-5.90)	0.07	3.09 (0.31-30.57)	0.33	0.90 (0.36-2.21)	0.81	1.16 (0.54-2.47)	0.70
		**Q4**	6.74 (1.86-24.48)	0.056	0.34 (0.08-1.36)	0.12	0.53 (0.22-1.23)	0.14	1.24 (0.58-2.64)	0.56
	**Adjusted**
		**Q1**	Reference		Reference		Reference		Reference	
		**Q2**	0.79 (0.25-2.42)	0.68	1.42 (0.26-7.64)	0.68	0.76 (0.29-1.94)	0.56	1.02 (0.45-2.32)	0.95
		**Q3**	2.17 (0.50-9.46)	0.29	6.28 (0.54-72.80)	0.14	0.69 (0.25-1.85)	0.46	1.58 (0.68-3.68)	0.28
		**Q4**	0.28 (0.08-0.94)	0.04	0.88 (0.17-4.64)	0.88	0.36 (0.13-1.04)	0.06	2.33 (0.91-5.99)	0.07

Data presented as OR (95%CI). Adjusted model: Adjusted for age, BMI, Education, Energy intake, Alcohol, Smoking, Vitamin-mineral use. Q: Quartile, Multivariable-adjusted odds ratio was used. SV: Semen volume, SC: Sperm concentration, TSM: Total sperm motility, NSM: Normal sperm morphology

**Table IV:** Abnormal semen quality across quartiles of dietary fat intake

**Variables**	**SC ˂ 20 M/ml**	**P-value**	**TSM ˂ 60%**	**P-value**	**NSM ˂ 4%**	**P-value**	**SV ˂ 3 ml**	**P-value**
**Cholesterol**
	**Crude**
		**Q1**	Reference		Reference		Reference		Reference	
		**Q2**	0.81 (0.33-1.98)	0.65	1.00 (0.23-4.18)	1.00	0.63 (0.27-1.47)	0.29	1.54 (0.72-3.27)	0.25
		**Q3**	4.21 (1.11-15.88)	0.03	2.06 (0.36-11.69)	0.41	1.00 (0.41-2.42)	1.00	0.92 (0.41-2.03)	0.84
		**Q4**	1.68 (0.61-4.66)	0.31	0.78 (0.20-3.07)	0.73	1.00 (0.41-2.42)	1.00	1.16 (0.54-2.51)	0.69
	**Adjusted**
		**Q1**	Reference		Reference		Reference		Reference	
		**Q2**	0.99 (0.39-2.53)	0.98	1.61 (0.36-7.18)	0.53	0.60 (0.25-1.45)	0.26	1.80 (0.81-3.98)	0.14
		**Q3**	5.63 (1.39-22.82)	0.01	4.13 (0.65-26.28)	0.13	0.93 (0.36-2.41)	0.88	1.14 (0.48-2.69)	0.75
		**Q4**	2.57 (0.78-8.42)	0.11	2.22 (0.44-11.18)	0.33	0.92 (0.33-2.53)	0.87	1.62 (0.66-3.97)	0.28
**SFA**
	**Crude**
		**Q1**	Reference		Reference		Reference		Reference	
		**Q2**	1.00 (0.4-2.50)	1.00	2.06 (0.36-11.69)	0.41	0.63 (0.27-1.47)	0.29	0.69 (0.33-1.47)	0.34
		**Q3**	3.10 (0.93-10.33)	0.06	1.00 (0.23-4.18)	1.00	1.00 (0.41-2.42)	1.00	0.64 (0.30-1.37)	0.25
		**Q4**	1.45 (0.54-3.88)	0.45	0.78 (0.20-3.07)	0.73	1.00 (0.41-2.42)	1.00	0.64 (0.30-1.37)	0.25
	**Adjusted**
		**Q1**	Reference		Reference		Reference		Reference	
		**Q2**	1.27 (0.48-3.34)	0.31	3.54 (0.58-21.29)	0.16	0.63 (0.26-1.52)	0.31	0.76 (0.34-1.65)	0.49
		**Q3**	4.82 (1.30-17.87)	0.01	2.34 (0.48-11.42)	0.29	0.96 (0.35-2.59)	0.94	0.71 (0.30-1.68)	0.43
		**Q4**	3.50 (0.84-14.60)	0.08	5.44 (0.74-39.93)	0.09	0.95 (0.27-3.27)	0.94	0.80 (0.27-2.34)	0.69
**Mufa**
	**Crude**
		**Q1**	Reference		Reference		Reference		Reference	
		**Q2**	1.00 (0.38-2.59)	1.00	0.73 (0.15-3.43)	0.69	1.09 (0.47-2.56)	0.82	0.81 (0.39-1.68)	0.57
		**Q3**	1.50 (0.53-4.23)	0.43	1.52 (0.24-9.43)	0.65	1.09 (0.47-2.56)	0.82	0.64 (0.30-1.37)	0.25
		**Q4**	1.50 (0.53-4.23)	0.43	0.47 (0.11-1.99)	0.30	1.09 (0.47-2.56)	0.82	0.54 (0.25-1.18)	0.12
	**Adjusted**
		**Q1**	Reference		Reference		Reference		Reference	
		**Q2**	1.33 (0.48-3.65)	0.57	1.21 (0.24-5.91)	0.81	1.07 (0.43-2.63)	0.87	0.82 (0.37-1.80)	0.63
		**Q3**	2.48 (0.75-8.17)	0.13	3.79 (0.52-27.47)	0.18	1.02 (0.38-2.73)	0.96	0.63 (0.26-1.52)	0.30
		**Q4**	3.93 (0.83-18.53)	0.08	2.93 (0.34-24.90)	0.32	0.98 (0.28-3.35)	0.97	0.54 (0.17-1.66)	0.28
**Pufa**
	**Crude**
		**Q1**	Reference		Reference		Reference		Reference	
		**Q2**	1.50 (0.53-4.23)	0.43	0.73 (0.15-3.43)	0.69	1.29 (0.57-2.90)	0.53	0.46 (0.21-1.02)	0.05
		**Q3**	1.00 (0.38-2.59)	1.00	3.09 (0.31-30.57)	0.33	1.73 (0.74-4.07)	0.20	0.55 (0.26-1.18)	0.13
		**Q4**	1.50 (0.53-4.23)	0.43	0.40 (0.09-1.62)	0.20	1.73 (0.74-4.07)	0.20	0.81 (0.39-1.68)	0.58
	**Adjusted**
		**Q1**	Reference		Reference		Reference		Reference	
		**Q2**	1.78 (0.60-5.24)	0.29	1.04 (0.21-5.15)	0.95	1.41 (0.60-3.30)	0.42	0.52 (0.23-1.18)	0.12
		**Q3**	1.34 (0.45-3.95)	0.58	5.98 (0.54-65.28)	0.14	2.07 (0.79-5.40)	0.13	0.70 (0.30-1.66)	0.42
		**Q4**	2.63 (0.63-10.83)	0.18	1.39 (0.20-9.49)	0.73	2.41 (0.75-7.73)	0.13	1.25 (0.46-3.40)	0.65
**Linoleic acid**
	**Crude**
		**Q1**	Reference		Reference		Reference		Reference	
		**Q2**	1.82 (0.62-5.34)	0.27	0.75 (0.16-3.49)	0.71	1.59 (0.69-3.68)	0.27	0.36 (0.15-0.82)	0.01
		**Q3**	0.92 (0.36-2.35)	0.87	3.19 (0.3-31.56)	0.32	1.47 (0.64-3.35)	0.35	0.72 (0.34-1.50)	0.38
		**Q4**	1.53 (0.54-4.31)	0.41	0.40 (0.10-1.65)	0.20	1.77 (0.75-4.16)	0.18	0.91 (0.44-1.88)	0.80
	**Adjusted**
		**Q1**	Reference		Reference		Reference		Reference	
		**Q2**	2.04 (0.67-6.18)	0.20	1.01 (0.21-4.87)	0.98	1.70 (0.71-4.02)	0.22	0.42 (0.18-1.00)	0.05
		**Q3**	1.88 (0.41-3.40)	0.74	6.35 (0.58-69.37)	0.12	1.69 (0.67-4.25)	0.26	1.04 (0.45-2.39)	0.92
		**Q4**	2.39 (0.60-9.46)	0.21	1.40 (0.21-9.28)	0.72	2.24 (0.72-6.95)	0.16	1.70 (0.64-4.52)	0.28
**Linolenic acid**
	**Crude**
		**Q1**	Reference		Reference		Reference		Reference	
		**Q2**	1.03 (0.38-2.81)	0.94	0.76 (0.16-3.55)	0.73	1.50 (0.64-3.50)	0.34	0.66 (0.30-1.40)	0.28
		**Q3**	0.93 (0.35-2.47)	0.89	0.77 (0.16-3.60)	0.74	1.53 (0.65-3.56)	0.32	0.69 (0.33-1.47)	0.34
		**Q4**	1.66 (0.55-4.98)	0.36	0.77 (0.16-3.60)	0.74	1.38 (0.60-3.18)	0.43	0.69 (0.33-1.47)	0.34
	**Adjusted**
		**Q1**	Reference		Reference		Reference		Reference	
		**Q2**	1.10 (0.40-3.03)	0.84	0.90 (0.18-4.29)	0.89	1.49 (0.63-3.50)	0.35	0.68 (0.31-1.47)	0.33
		**Q3**	1.14 (0.40-3.24)	0.79	1.60 (0.30-8.42)	0.57	1.55 (0.63-3.78)	0.33	0.80 (0.36-1.77)	0.58
		**Q4**	2.45 (0.67-8.95)	0.17	3.07 (0.45-20.81)	0.25	1.40 (0.52-3.75)	0.50	0.88 (0.36-2.15	0.78

Data presented as OR (95% CI). Adjusted model: Adjusted for age, BMI, Education, Energy intake, Alcohol and smoking, Vitamin-mineral use. Q: Quartile, SFA: Saturated fatty acid, Mufa: Mono-unsaturated fatty acid, Pufa: Poly-unsaturated fatty acid. Multivariable-adjusted odds ratio was used for analysis. SV: Semen volume, SC: Sperm concentration, TSM: Total sperm motility, NSM: Normal sperm morphology

## 4. Discussion

In this cross-sectional study on 260 infertile men, we found that calcium intake was reversely correlated with SC and positively related to NSM. Furthermore, folate intake showed a significant relationship with SC and morphology and there was a significant relationship between SC and selenium intake. In addition, there was a reverse association between cholesterol and SFA intake with SC.

The results of recent studies have both been in favor of, and contradict, the results of the present study. The possible effect of dairy foods and calcium on male fertility is highly controversial. Unlike the present study, the results of other studies have found no association between blood calcium concentration, the time of first serving, and the time of withdrawal from pregnancy, respectively (16, 17). In addition, one study found that serum calcium concentrations were in contrast to Ca ${}^{2+}$ concentrations, which could be affected by blood pH and protein concentrations (16). Also, in plasma, calcium is available in 3 forms: ionized, complex, and bound protein. Among these, calcium ionization (Ca ${}^{2+}$ ) is the physiologically active form, so measuring plasma Ca ${}^{2+}$ concentration, as opposed to total plasma concentration, is an indicator of physiologically present calcium (18). In another study, no significant relationship was observed between calcium and sperm parameters (19). However, high-fat dairy products and cheese have been inversely related to sperm quality parameters (20), while low fat milk and skimmed milk were associated with better classical semen indicators. Consumption of low-fat and skimmed milk can be associated with higher levels of insulin and insulin-like growth factor-1 and thus spermatogenesis, while the reason for the adverse effect of high-fat dairy on infertility can be ascribed to the high content of SFA (21).

As seen in our study, selenium intake indicated a preservative effect against SC. For example, some minerals such as selenium and zinc may develop semen quality via their anti-inflammatory and protective roles against free radicals (22). There is a direct relation between the antioxidant status and the production of reactive oxygen species (ROS) in sperm (23). In addition, high concentrations of ROS negatively affect sperm DNA and, in turn, impact sperm motility, vitality and concentration, as well as potentially contributing to miscarriages and developmental abnormalities in offspring (24). Antioxidants are considered as `scavengers' of ROS and their use has been studied as a treatment to neutralize the negative effect of high concentrations of ROS on semen parameters. A recent study also indicated the importance of selenium in completing sperm maturation and quality (25). Selenium, as a selenoprotein, is more involved in male reproductive function. Adequate selenium intake is important for normal spermatogenesis and sperm maturation (26).

Additionally, folate intake displayed significant correlations with SC and morphology in our study. In men with high folate intake, lower levels of aneuploid sperm have been reported, suggesting that this vitamin (found mainly in green leafy vegetables) may play a role in spermatogenesis due to its role in DNA retention, RNA transport, and protein synthesis (27). In addition, the beneficial effects of dietary antioxidants such as vitamin C, vitamin E, and folate on sperm motility have already been shown. The results of a randomized controlled trial showed that after treatment with sulfate and folic acid, the number of normal sperm increased in infertile and fertile men (28). In a clinical trial, analysis of DNA methylation in sperm with exposure to folic acid suggested the susceptibility of mice and humans in sequences of potential importance to germ cells and embryonic growth. This study also suggested hypomethylation of sperm DNA and involvement in the regulation of Methyl tetrahidrofolate reductase as a possible mechanism for folic acid supplementation for different species (29).

Furthermore, supporting the findings of our study, a previous study found an inverse relationship between dietary fat, especially cholesterol and SFA intake and SC. Trans-fatty acids, SFAs, and preservatives or hormonal residues such as xenobiotic or anabolic steroids may alter sperm quality (30). High SFA concentrations and low omega-3, poly-unsaturated fatty acids (PUFA) levels are associated with decreased fertility parameters (19). In an animal study, some dietary SFAs did not affect sperm quality parameters. However, one human study showed that higher levels of palmitic acid or stearic acid were present in the sperm of infertile men (31). Also, in our study, linoleic acid intake showed some preservative effects on sperm volume in the second quartile of intake. Several mechanisms have been proposed for the beneficial effects of PUFA and MUFA on sperm quality. Adipose tissue plays an important role in causing oxidative stress after an inflammatory disorder that may alter normal reproductive pathways and sperm activity (32, 33). In fact, under stress conditions, mitochondria are likely to cause a cascade of oxidative damage in the testicular environment. MUFA and PUFA fatty acids have protective effects against increased ROS levels due to their anti-inflammatory and antioxidant properties (34, 35).

### Limitations

The present study, like other cross-sectional studies, is not able to definitively determine the cause-and-effect relationship between the variables and it is suggested that clinical trial or case-control studies be performed to confirm the results. Another limitation is that plasma or semen levels of minerals and fatty acids were not considered in this study. Finally, the 168-item food frequency questionnaire has some limitations in recording food intakes and relies on the individual's memory, and is not always as accurate when employed by the elderly or illiterate people; therefore, the use of 3-day food recall could be useful in future studies.

## 5. Conclusion

The results of the present study showed that high calcium intake was inversely related to SC and directly related to normal morphology. Also, folate and selenium intake showed protective effects on SC. On the other hand, the high levels of cholesterol and SFA intake showed an inverse relationship with SC.

##  Conflict of Interest

The authors declare that there is no conflict of interest.
